# Local recurrence of melanocytoma of the cervical spine

**DOI:** 10.1007/s13760-023-02459-9

**Published:** 2024-02-06

**Authors:** Eleanor M. Moncur, Selma Al-Ahmad, Maria Thom, Claudia L. Craven, David Choi

**Affiliations:** 1 Victor Horsley Department of Neurosurgery, National Hospital for Neurology and Neurosurgery, Queen Square, London, WC1N 3BG UK; 2 Department of Neuropathology, Institute of Neurology, Queen Square, London, WC1N 3BG UK; 3 Department of Neurosurgery, Cambridge University Hospitals, Hills Road, Cambridge, CB2 0QQ UK

**Keywords:** Melanocytoma, Cervical spine, Recurrence, Histopathology, Magnetic resonance imaging

## Introduction

Melanocytomas are rare and can mimic various pathologies. We present an illustrative report to describe the key radiological and histological features to diagnose and differentiate intradural melanocytomas.

## Clinical description

**Fig. 1:**
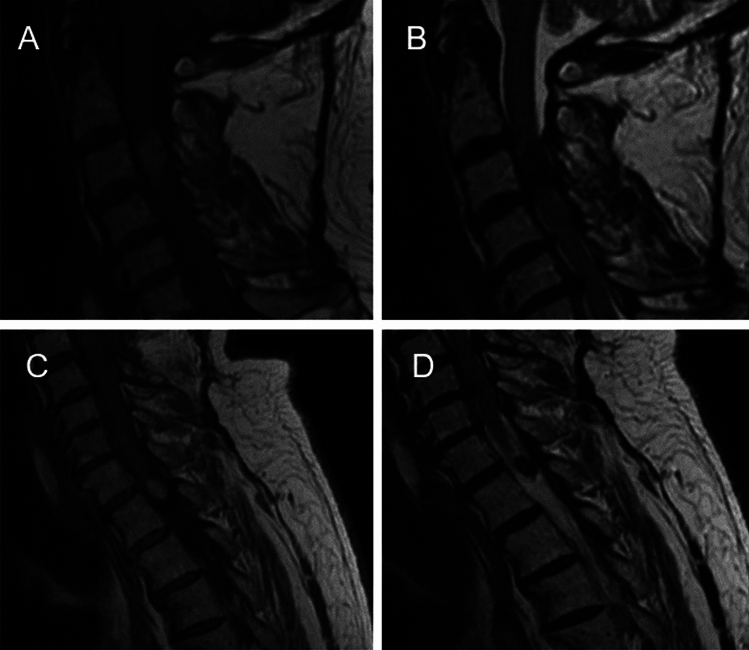
**A** T1-weighted image of C3 lesion. **B** T2-weighted image of C3 lesion. **C** T1-weighted image of T1 (vertebral level) lesion **D** T2-weighted image of T1 (vertebral level) lesion showing lesions were hyperintense on T1-weighted, and hypointense on T2-weighted MR imaging

This septuagenarian underwent investigations for worsening clumsiness in his hands and ataxic gait. MRI showed an intradural cervical (C3) lesion and a smaller lesion at the first thoracic (T1) level (Fig. 1A–D). These lesions were hyperintense and hypointense on T1 and T2 weighted MRI respectively. Computed tomography and full clinical examination demonstrated no other primary lesions. The cervical lesion was excised in 2007, and the smaller T1 lesion was excised in 2009 and then re-excised in 2011 after local recurrence.

**Fig. 2:**
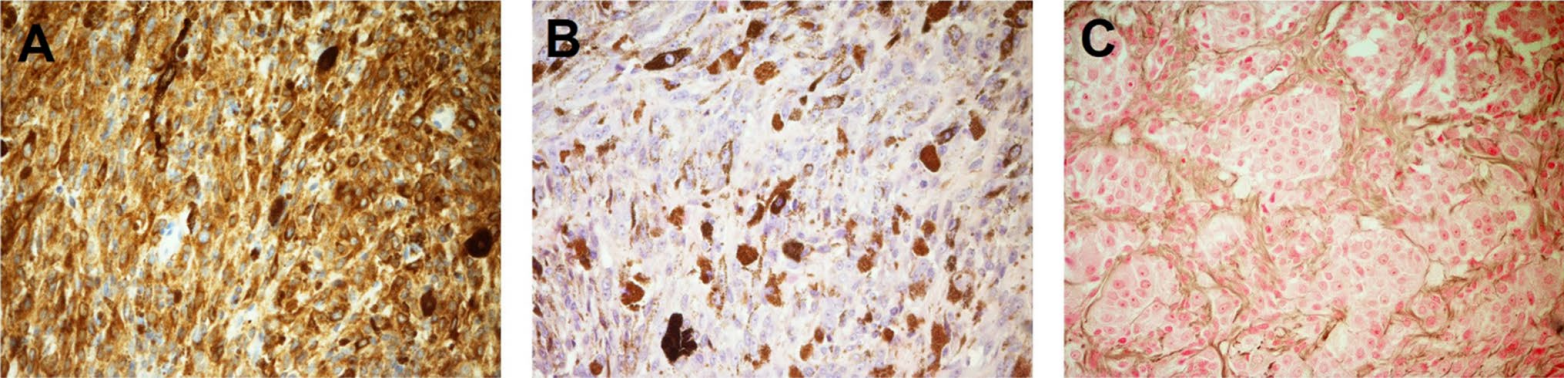
**A** Hematoxylin and eosin stain showing plump spindle cells, minimal mitotic activity and melanin pigment. B Immunocytochemistry for HMB45 protein. **C** Bleached section showing bland cytology, nested architecture and absence of mitotic activity (low Ki67 index). All images were taken at × 20 magnification

Histopathology from the first samples showed plump spindle and epithelioid cells which stained positive for Melan-A (Fig. 2A) and HMB45 (Fig. 2B), with intracytoplasmic granular black pigment. The tissue also had unremarkable nuclei, low mitotic activity and low Ki67 with no haemorrhage or necrosis, consistent with a primary intradural melanocytoma (Fig. 2C).

## Discussion

Primary intradural melanocytomas are rare, low-grade, pigmented lesions of the central nervous system (CNS) originating from melanocytes within the leptomeninges [1]. When occurring in the spine, they have a predilection for the cervical spine as extramedullary intradural lesions [2].

In terms of histology, melanocytomas have spindle, fusiform or polygonal-shaped cells with variable amounts of melanin pigment in cytoplasm [1, 3, 4]. Like melanomas, they stain for melan-A and HMB45. They differ from melanoma in that they have no evidence of anaplasia, no mitotic figures and are usually not necrotic or hemorrhagic. Oncogenic mutations GNAQ or GNA11 are common in melanocytomas.

Melanocytomas lack distinctive imaging characteristics due to variability in the amount of melanin present [1]. However, most reports describe lesions which are well defined, iso or hyperintense signals T1-weighted images and hypointense signals on T2-weighted images [4]. Unlike meningiomas, melanocytomas heterogeneously enhance with contrast [4].

The main histological differentials include other pigmented neoplasms such as melanoma or melanotic schwannoma and less often, meningioma, ependymoma, glioma or metastases [1, 2].

Due to their propensity for recurrence, management is aimed at gross total resection [1]. Although classified as low grade, lesions can transform to melanoma and recurrence is common, even after excision [1, 4]. Therefore, close follow-up with serial MRI is important in monitoring residual tumour or recurrence and to determine if early adjuvant radiotherapy or re-resection is needed [1, 2]. Options after resection include adjuvant radiotherapy or surveillance monitoring (if the patient is asymptomatic) [2, 5].

## Conclusion

We describe the key radiological and histological features to diagnose and differentiate intradural melanocytomas. Cervical melanocytomas can mimic both high- and low-grade lesions, such as meningiomas and melanomas. Although low grade, management is aimed at gross total resection due to the high chance of local recurrence and or transformation.
